# Analysis of Macroporous Resin Combined Extraction and Purification of Polyphenols from *Agrimonia pilosa* Ledeb. and Anti-Tumor Effect In Vitro

**DOI:** 10.3390/molecules30071478

**Published:** 2025-03-26

**Authors:** Zhanghao Mei, Huifen Li, Tingting Li, Huiping Taoli, Linmei Pan

**Affiliations:** 1 Department of Pharmacy, Nanjing University of Chinese Medicine, Nanjing 210023, China; 13770692667@163.com (Z.M.); 18943092260@163.com (H.L.); 18348285155@163.com (T.L.); 2 Jiangsu Research Center of Botanical Medicine Refinement Engineering, Nanjing 210023, China; 3 Jiangsu Collaborative Innovation Center for Tumor Prevention and Treatment with Traditional Chinese Medicine, Nanjing 210023, China; tlhp1109@njucm.edu.cn

**Keywords:** *Agrimonia pilosa* Ledeb., polyphenols, macroporous resin purification, liquid–liquid extraction, quantitative analysis, anti-tumor effects

## Abstract

*Agrimonia pilosa* Ledeb. (APL), a traditional Chinese herb frequently employed by Professor Zhou Zhongying, a master of traditional Chinese medicine, for colorectal cancer treatment, is rich in polyphenols with potential anti-tumor properties. To elucidate its bioactive components, this study developed a two-step purification process combining macroporous resin adsorption and liquid–liquid extraction to enrich polyphenols from APL (APLs). The adsorption/desorption mechanisms of APLs on macroporous resins were systematically investigated through resin screening, adsorption kinetics, and thermodynamics. The Langmuir isotherm model confirmed the adsorption process as spontaneous and exothermic. Pseudo-second-order kinetics effectively described the adsorption behavior of D101 resin. Optimized adsorption and column elution parameters were established, followed by liquid–liquid extraction for further purification. The components were compared and analyzed by ultra-performance liquid chromatography and quadrupole time-of-flight tandem mass spectrometry (UPLC-Q-Zeno-TOF-MS/MS). It was preliminarily identified that 29 polyphenols were mainly concentrated in water-saturated n-butanol (BEA) and ethyl acetate (ECA) extract fractions. Quantitative analysis using ultra-high performance liquid chromatography–triple quadrupole liquid chromatography–mass spectrometry (UHPLC-C-QTRAP-MS/MS) revealed higher contents of catechin (66.67 ± 1.33 ng·mg^−1^), hyperoside (382.56 ± 3.65 ng·mg^−1^), and chlorogenic acid (10.60 ± 0.05 ng·mg^−1^) in BEA compared to ECA (46.00 ± 2.00, 239.40 ± 2.60, and 3.42 ± 0.01 ng·mg^−1^, respectively). In vitro experiments demonstrated that BEA exhibited superior antiproliferative activity (IC_50_: 434.5 μg·mL^−1^) and significantly inhibited CT26 tumor cell migration compared to ECA (IC_50_: 672.5 μg·mL^−1^). The enhanced biological activity of BEA may be due to its higher polyphenol content, suggesting that these compounds mediate their anti-tumor effects through different biochemical pathways. This work lays the foundation for exploring the multi-target mechanism of anti-tumor effects of APLs.

## 1. Introduction

First documented in the Ben Cao Tu Jing (Song Dynasty), *Agrimonia pilosa* Ledeb. (APL) is a perennial herb of the Rosaceae family. Recognized for its astringent hemostatic, detoxifying, and deficiency-tonifying properties, APL constitutes the most frequently utilized herb in the prescriptions of Zhou Zhongying, a master of traditional Chinese medicine, for colorectal cancer treatment [1]. In the clinical medication of 1374 patients with malignant tumors, the frequency of APL use was as high as 63.32 [2]. Based on Master Zhou Zhongying’s theory of cancer toxin pathogenesis and clinical prescription, Professor Cheng Haibo formed Xianlian Jiedu Decoction with APL as the main herb and verified that it can significantly inhibit the proliferation of colorectal cancer cells and induce apoptosis [3]. Another study investigated the effect of the *Agrimonia pilosa* Ledeb.—Coptis chinensis herb pair on colorectal cancer, mainly by inhibiting the PI3K/AKT pathway [4]. Also, the compound preparation Changyan Capsule, with APL as its primary component, has been clinically employed in managing acute/chronic enteritis induced by spleen deficiency with damp-heat pathogenesis [5,6].

Current investigations have identified polyphenolic compounds as the predominant phytochemical constituents in APL, especially in terms of their anti-tumor properties with multiple potential therapeutic effects [7]. Cerezo-Guisado et al. [8] found that catechin can induce cell cycle arrest and promote apoptosis of colorectal cancer tumor cells by inhibiting the activation of Akt and ERK1/2 signaling pathways. Chlorogenic acid induces the production of reactive oxygen species and inhibits the activity of human colorectal cancer cells [9]. Hyperoside inhibits tumor growth in tumor-bearing mice by inducing apoptosis through caspase-3 and NF-κB signaling pathways [10]. Hnit et al. also confirmed that agrimonyol B in APL can block the cell cycle progression of cancer cells by G0 phase arrest [7].

Emerging evidence highlights the pharmacologically significant synergism among phytoconstituents [11]. Notably, combinatorial interactions between polyphenolic monomers amplify therapeutic outcomes [12]: quercetin and resveratrol cooperatively downregulate inducible nitric oxide synthase (iNOS) gene expression, effectively reducing macrophage-derived NO production and potentiating anti-angiogenic effects [13,14]. Catechin demonstrates collaborative antioxidant and anti-inflammatory actions with quercetin, gallic acid, and curcumin, collectively delaying colorectal carcinogenesis [15,16,17]. A 1:1 ratio of quercetin to hyperoside exhibits enhanced anti-cancer activity through miR-21 signaling pathway inhibition [18]. These findings systematically elucidate the synergistic network underlying APL’s polypharmacological effects, providing a molecular rationale for its traditional application in cancer and inflammatory disorders.

The premise of clarifying its anti-tumor mechanism is to correspond to the relevant material basis. Therefore, optimizing the appropriate separation methods to obtain and analyze the types and contents of various components is of great significance for elucidating their multi-target synergistic anti-tumor effects. Current purification techniques for polyphenols primarily encompass solvent extraction, macroporous resin adsorption, high-speed counter-current chromatography, membrane separation, and supercritical fluid extraction [19,20]. Among these, macroporous resin adsorption has emerged as a predominant methodology in traditional Chinese medicine component purification (particularly for polyphenols and alkaloids) due to its operational simplicity, high selectivity, and ease of regeneration [21,22,23]. The resin selection process necessitates systematic consideration of multiple critical factors: polarity matching between resin matrix and target polyphenols, as well as a comprehensive evaluation of physical–chemical characteristics such as surface area, pore diameter distribution, and chemical stability. Specifically, adsorption isotherms and kinetic modeling were used to determine the adsorption capacity and equilibrium time, whereas thermodynamic parameter analyses provided insights into the energy variations during the adsorption process [24]. This multidimensional analytical approach facilitates the scientific screening of optimal macroporous resins with superior adsorption performance and cost-effectiveness.

Although macroporous resin chromatography has been employed for the purification of APL extracts, the resulting macroporous resin-enriched products exhibit relatively low purity. Conventional purification techniques for natural products typically involve liquid–liquid extraction, fractional distillation, and silica gel column chromatography [25]. APLs, characterized by their polyhydroxy structure, interact with impurities through intermolecular hydrogen bonding and other molecular interactions. Solvent extraction employing organic solvents has demonstrated potential for further impurity removal through selective dissolution. Previous studies have reported enhanced purification efficiency for ginsenosides [26] and baicalin [27] through the synergistic combination of macroporous resin adsorption and solvent extraction. However, no existing literature documents the application of integrated macroporous resin–liquid–liquid extraction techniques for polyphenol isolation from APL.

This study aims to establish an efficient and practical protocol for APL purification. We propose a combinatorial purification strategy employing optimized macroporous resin adsorption coupled with liquid–liquid extraction. The chemical profiles of different fractions will be characterized using liquid chromatography–mass spectrometry (LC-MS) for both qualitative and quantitative analysis. Comparative evaluation of antiproliferative efficacy against CT26 colon carcinoma cells and inhibitory effects on cellular migration will be conducted through in vitro experiments to identify the optimal purification parameters. This systematic approach will enable the development of a standardized purification protocol for obtaining bioactive polyphenolic compounds from APL.

## 2. Results

### 2.1. Results of Macroporous Resins

The adsorption and desorption characteristics of macroporous resin are affected by surface area, pore size, polarity of adsorbent, structure and properties of adsorbate [28]. Considering the polyhydroxy structure of polyphenols, six resins with different surface areas and pore sizes (from polar to non-polar) were selected as adsorbents. The relevant information is in the Supplementary Material Table S1.

**Figure 1:**
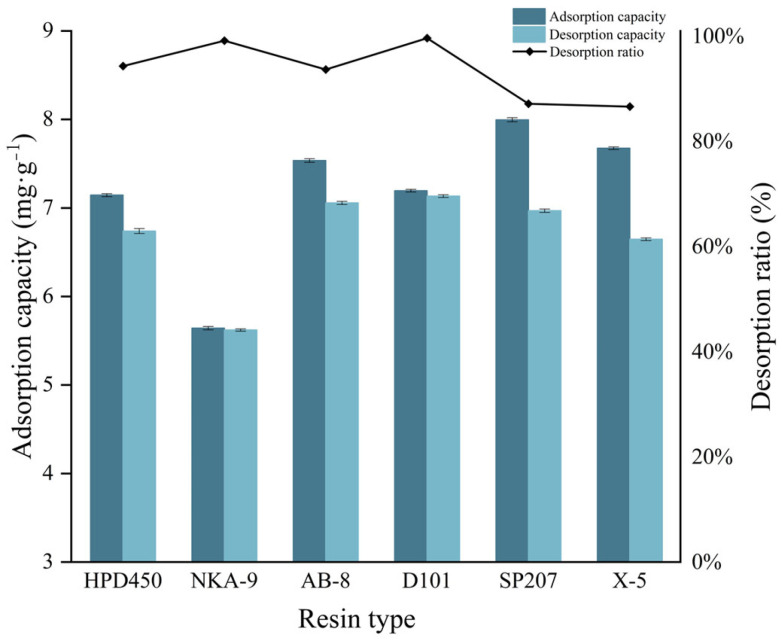
Results of adsorption/desorption capacities and desorption ratios of six resins for APLs.

As shown in Figure 1, the adsorption/desorption capacity of six macroporous resins was tested. The results showed that the order of adsorption capacity from large to small was SP207 > X-5 > AB-8 > D101 > HPD450 > NKA-9. With the decrease of polarity, the adsorption capacity showed an upward trend. In addition to the small surface area of NKA-9, the others were relatively large, which may be related to the surface area. From the perspective of desorption rate, the highest is D101, the lowest is X-5, but its pore size is the largest, indicating that the surface area is not the only influencing factor, and the results need to be comprehensively analyzed [28]. In the order of desorption capacity, D101 and SP207 were higher.

In addition, due to the influence of hydrophobic interactions, van der Waals forces and other factors, macroporous resins have a wide range of selectivity for polyphenols containing multiple polar groups. Among them, the polarity of D101 resin is moderate, and its benzene ring structure produces a moderate hydrophobic interaction with the aromatic rings of polyphenols through π-π conjugation, while residual polar groups can form weak hydrogen bonds. Although the adsorption capacity of SP207 was slightly higher than that of D101, the selection index of macroporous resins should also consider the overall desorption rate and desorption capacity. The desorption rate and desorption capacity of D101 macroporous resin were higher than those of SP207. It is speculated that macroporous D101 resin may be more suitable for the structure of the target polyphenols due to its polarity and pore size, thus achieving a balance between adsorption and desorption. Therefore, this experiment selected D101 as the adsorbent for subsequent experiments.

### 2.2. Adsorption Dynamics

**Figure 2:**
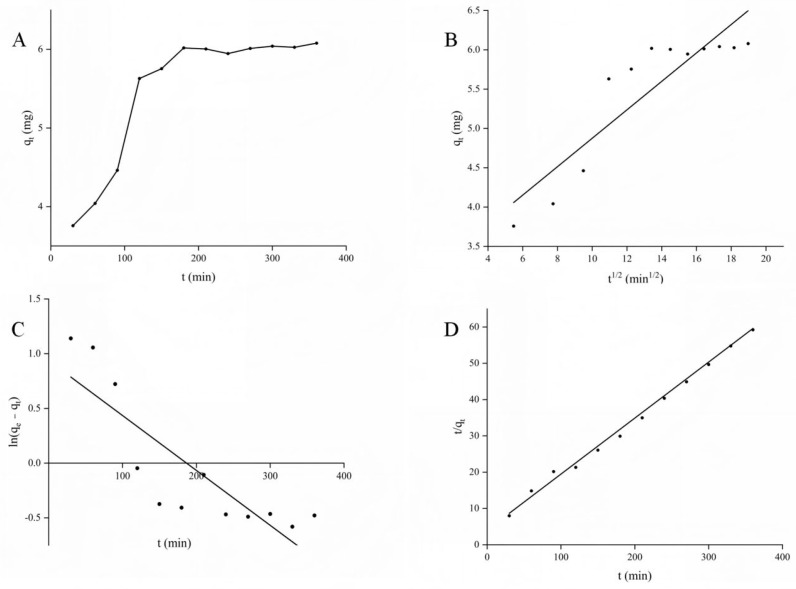
(**A**) adsorption kinetics curve, and linear fitting of total polyphenols on D101 resin at 25 °C based on (**B**) intraparticle diffusion, (**C**) pseudo-first-order kinetics and (**D**) pseudo-second-order kinetic models.

The adsorption kinetics of APLs on D101 macroporous resin was investigated at 25 °C, and as shown in Figure 2A, the adsorption of APLs on D101 macroporous resin increased rapidly during the initial time (120 min), after which the growth rate slowed down, approached a plateau, and reached the equilibrium point (180 min). This may be due to the fact that at the beginning of the adsorption, there were enough adsorption sites on D101 resin, and there was a concentration difference in APLs between the solution and the resin, whereas as the adsorption proceeded, more and more adsorption sites were occupied by APLs and other components until adsorption reached equilibrium.

The experimental data were fitted to the pseudo-first-order, pseudo-second-order, and intra-particle diffusion models [29,30], and the results are shown in Figure 2B–D. Related experimental parameters are provided in Table S2 of the Supplementary Material. For the pseudo-first-order model and the pseudo-second-order model, comparing the values of the correlation coefficient R^2^ shows that the pseudo-second-order model (R^2^ = 0.9952) is better than the pseudo-first-order model (R^2^ = 0.7235). Additionally, the theoretical maximum adsorption amount obtained from the pseudo-second-order model (q_2_ = 6.6243 mg·g^−1^) is closer to the actual adsorption amount (q_e_ = 6.0182 mg·g^−1^) than that of the pseudo-first-order model (q_1_ = 6.8944 mg·g^−1^). This suggests that the adsorption of total polyphenols of APL on D101 follows the pseudo-second-order model, indicating that there are many factors limiting the rate of adsorption [31].

From the results of the intra-particle diffusion model fitting, the diffusion curves of q_t_ versus t^1/2^ show a multilinear relationship with increasing adsorption time, and the adsorption process can be divided into three stages [32]. The first stage (0–10 min) exhibits boundary layer diffusion when diffusion occurs from the solvent to the surface of the resin particles; the second stage (120–180 min) is a gradual adsorption stage where internal diffusion is rate-limiting; and the third stage (210–360 min) is the final equilibrium stage. It is shown that the process is controlled by multiple diffusion processes. The results of intra-particle diffusion confirmed the conclusion of pseudo-second-order kinetics, suggesting that there may be many factors limiting the adsorption of APLs on macroporous resin.

### 2.3. Adsorption Isotherm

**Figure 3:**
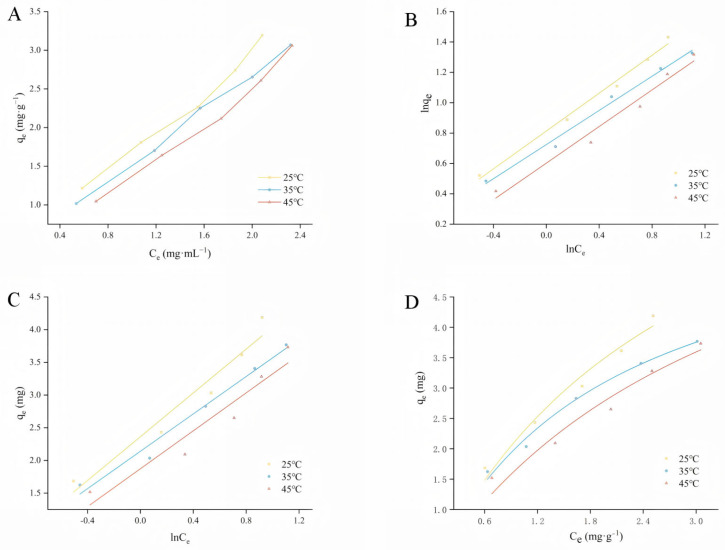
Three models for total polyphenols on D101 resin at 25, 35, and 45 °C: (**A**) adsorption isotherms, (**B**) Freundlich model, (**C**) Temkin model, and (**D**) Langmuir model.

The adsorption isotherm characterizes the relationship between the equilibrium amount of adsorbate adsorbed on the resin and the equilibrium concentration of the unadsorbed adsorbate in solution when equilibrium is reached at a certain temperature [33]. In this study, adsorption at three temperature gradients (25 °C, 35 °C, and 45 °C) was simulated. As can be seen in Figure 3, the adsorption of D101 decreases with increasing temperature at the same concentration, indicating that high temperatures are unfavorable for the adsorption of APLs. The adsorption process was modeled using the Langmuir, Freundlich, and Temkin adsorption isotherm models.

Among them, the Langmuir model describes an adsorption process on a single molecular layer, with no interaction between the adsorbed molecules. The Freundlich model can be used to account for both unimolecular and multimolecular layer adsorption, and the Temkin model takes into account the interaction between the adsorbent and the adsorbed substance in a linear mode [34]. The high correlation coefficients indicate that these models are suitable for describing the tested adsorption systems within the range of concentrations studied, whereas the correlation coefficients of the Temkin model are greater than 0.9, which indicates that the adsorption of D101 resin may involve some degree of multilayer adsorption. K_T_ shows a trend of decreasing with temperature, indicating that high temperature is not conducive to the adsorption of D101 resin by APLs solution.

Where K_L_ is Langmuir’s constant, K_L_ (0 < K_L_ < 1) indicates that the isotherm is favorable [35]. This experimental result is similar to the findings of Wang et al. [36]. While K_F_ is a Freundlich affinity parameter for heterogeneous dispersed systems, n is related to the size, which in turn is related to the adsorption driving force and the energy distribution of the adsorption sites. Adsorption is difficult to occur when the value of 1/n is between 0.5 and 1 [35]. Therefore, by comparing the correlation coefficients of the above models (shown in Table S3 of the Supplementary Material), it can be concluded that these models were used for the adsorption of APL solutions onto D101 macroporous resin at 25 °C.

### 2.4. Adsorption Thermodynamics

**Figure 4:**
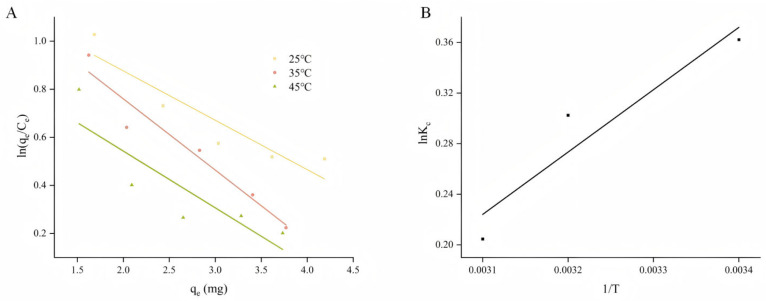
(**A**) Linear fitting of ln (qe/Ce) and qe at different temperatures and (**B**) linear fitting of Kc and 1/T. q_e_ refers to equilibrium adsorption capacity; C_e_ refers to equilibrium concentration; T refers to temperature; and K_c_ refers to equilibrium constant.

Adsorption thermodynamics can provide additional theoretical information to shed further light on changes in energy and adsorption mechanisms. The K_c_ values were calculated by fitting a linear equation with q_e_ and ln (q_e_/C_e_) as the horizontal and vertical coordinates. The intercept of the fitted linear equation was K_c_ (Figure 4A). From the results of this experiment, it can be seen that K_c_ decreases with increasing temperature, ΔG < 0, indicating that the adsorption process is spontaneous. Additionally, the absolute value of ΔG decreases with increasing temperature, indicating that low temperatures are favorable for adsorption. This result is in agreement with that of the adsorption isotherm [37]. Using 1/T and ln K_c_ as horizontal and vertical coordinates (Figure 4B), ΔH and ΔS were calculated from the slopes and intercepts of the fitted linear equations in Table 1. An absolute value of ΔH (4.09 kJ·mol^−1^) < 0 indicated that the adsorption process was exothermic, and ΔS < 0 indicated that the stoichiometric nature of the adsorption of the polyphenols decreased [38].

### 2.5. Dynamic Adsorption and Desorption

#### 2.5.1. Dynamic Leakage Curve

**Figure 5:**
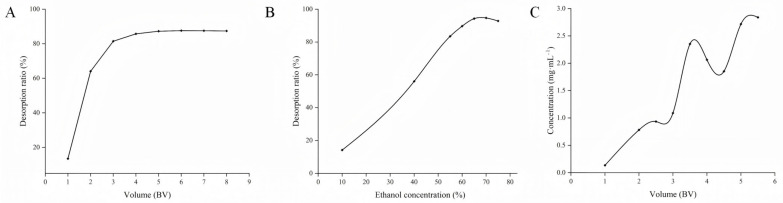
Effect of ethanol concentration on desorption capacity of D101 resin: (**A**) dynamic leakage curve; (**B**) effects of ethanol concentrations; and (**C**) ethanol elution volume. The dots in the figure are the results of data detection.

Dynamic leakage curves accurately reflect the adsorption behavior between the target adsorbent and the resin, as well as changes in the adsorption capacity of the resin. This information helps accurately determine important parameters such as the volume of the sample. Therefore, dynamic leakage curves play an important role in reducing the costs of the separation process. In this study, the leakage point is defined as the point at which the APLs in the elution reach approximately 1/10 of the initial concentration. Usually, the resin is unable to retain the target adsorbate at the breakthrough point and the adsorbate molecules begin to leak from the resin [38]. The initial polyphenol concentration was 18.68 mg·mL^−1^, and the injection was stopped when the effluent polyphenol concentration reached approximately 1.87 mg·mL^−1^. Figure 5A shows that the leakage point appeared at 3.5 BV (bed volume, volume of macroporous resin). Considering the actual production, the sample volume on the column was determined to be 4 BV.

#### 2.5.2. Selection of Eluting Agents and Dosage

APLs were desorbed in the macroporous D101 resin using different concentrations of ethanol (10%–80%) at a rate of 1 BV·h^−1^, and the concentration with optimal elution was determined by comparing the resolution rate of the adsorbent at various ethanol concentrations. Figure 5B shows that a resolution rate of 94.69% achieves the optimum separation when the ethanol concentration is 70%, while a further increase in ethanol concentration leads to a decreasing trend in the desorption rate. This phenomenon indicates that neither higher nor lower concentrations of ethanol are not favorable for polyphenol solubilization. Therefore, 70% ethanol was selected as the optimal eluent. Also, upon examining the eluent dosage, it was found (Figure 5C) that when the eluent dosage was greater than 5 BV, no more APLs were detected, so the final elution was carried out at a dosage of 5 BV of 70% ethanol.

### 2.6. Characterization of the Extraction Products from the Macroporous Resin of APL

**Figure 6:**
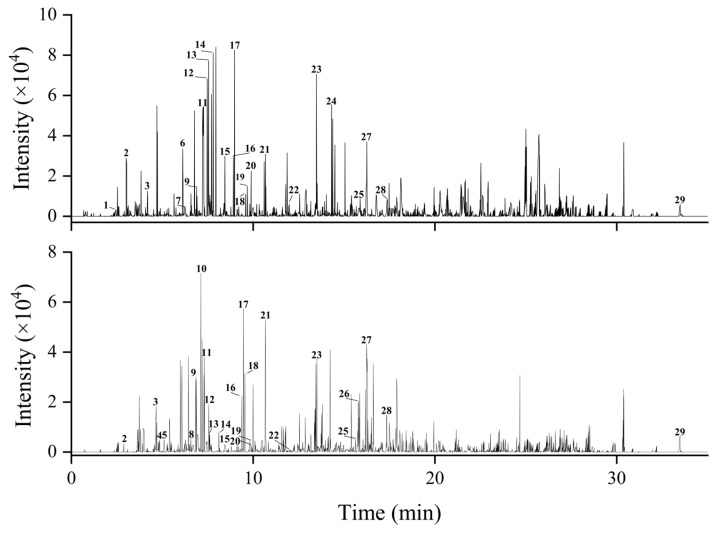
The ion current (BPC) diagram of (**A**) BEA extract and (**B**) ECA extract in negative ion mode after purification of APL. The numbers in the figure correspond to the various compounds in Table 2.

Polyphenols are widely and abundantly distributed and can be categorized into four main groups: phenolic acids, flavonoids, lignans, and stilbenes [39]. In this study, the phenolic acid and flavonoid components were tested in positive and negative ion modes using UPLC-Q-Zeno-TOF-MS/MS, and the data were analyzed using Peak View TM 1.2.1 software. It was found that the components in the total polyphenols of APL in water-saturated n-butanol (BEA) and ethyl acetate (ECA) were mainly concentrated in negative ion modes (Figure 6). The components of the two parts were identified based on mass spectrometry information, such as retention time and fragment ion peak from published data. A total of 29 kinds of purified macroporous resin refined products were identified (Table 2), including 12 kinds of phenolic acids, 14 kinds of flavonoids, and 3 kinds of other polyphenols. Their corresponding structures and CAS numbers are presented in the Supplementary Material, Table S4.

**Figure 7:**
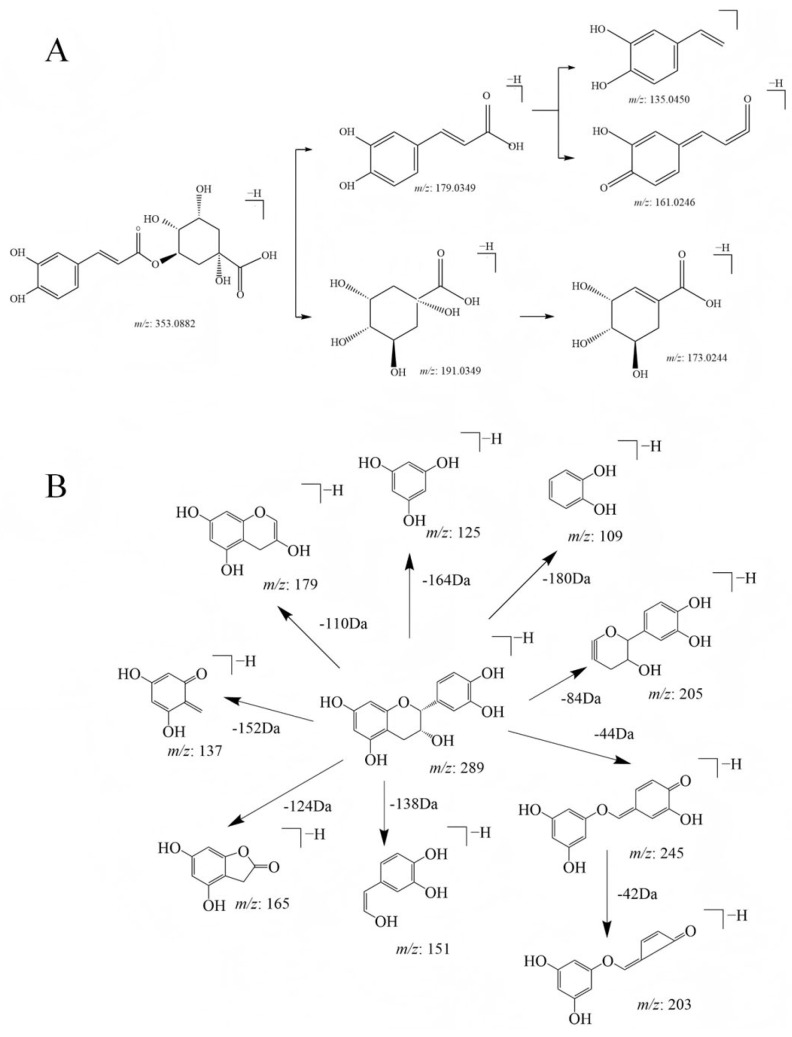
Cleavage pathways for two compounds: (**A**) the cleavage pathway of chlorogenic acid and (**B**) catechin.

Through the analysis conducted in positive ion mode, a total of 12 phenolic acid compounds were identified (compounds **1**, **2**, **4**, **6**–**9**, **11**, **16**, **19**, **20**, and **22**). Among the phenolic acid components, compound 16, for example, showed its [M-H]^−^ ion mass spectral peak at *m*/*z* 353.0882 with a retention time of 8.9 min. Comparative analysis of the retention time and mass spectral data of the control substance confirmed that the compound is chlorogenic acid, with the molecular formula C_16_H_18_O_9_. The cleavage process of chlorogenic acid with the molecular ion [M-H]^−^ at *m*/*z* 353.0882 (Figure 7A) was initially triggered by the loss of the caffeoyl group, followed by the generation of the MS^2^ fragment ion of quinic acid at *m*/*z* 191.0560 of acid and the further production of caffeic acid fragment ions at *m*/*z* 179.0349 through the additional loss of quinic acid [40].

A total of 14 flavonoids (compounds **3**, **5**, **12**–**15**, **17**, **18**, **21**, **23**–**27**) were identified in negative ion mode. Taking compound 3 as an example, the quasi-molecular ion peak *m*/*z* 289 [M-H]^−^, obtained via mass spectrometry and elemental composition analysis, suggests that the possible molecular formula of the compound is C_15_H_14_O_6_. Secondary mass spectrometry analysis was then carried out with *m*/*z* 289 [M-H]^−^ as the parent ion. The characteristic ions and their corresponding secondary mass spectral information are depicted in the Supplementary Material (Figure S1). The secondary fragment ions had obvious *m*/*z* 245 [M-H-C_2_H_4_O]^−^, *m*/*z* 205 [M-H- 84]^−^, *m*/*z* 203 [M-H-C_2_H_4_O-C_2_H_2_O]^−^, *m*/*z* 179 [M-H-C_6_H_6_O_2_]^−^, and *m*/*z* 109 [M-H-180]^−^. Among them, the fragment ion at *m*/*z* 245 was generated due to the loss of the C_2_H_4_O group. The fragment ion at *m*/*z* 205 was produced by the cleavage of the A ring. The ion at *m*/*z* 179 was due to the loss of the catechol group (C_6_H_6_O_2_). The loss of ring A and ring C produced *m*/*z* 109 ions. The ion with an *m*/*z* of 203 was due to the RAD cleavage. This cleavage rule was similar to the discovery of Mutungi et al. [41], so compound 3 was identified as catechin (Figure 7B).

### 2.7. UHPLC-C-QTRAP-MS/MS-Based Quantitative Analysis of the Macroporous Resin-Extracted Products of APL

#### 2.7.1. Methodological Validation

UHPLC-C-QTRAP-MS/MS was used to quantify the components with a better response in the macroporous resin-extracted products of APL. Some of the compounds were analyzed using an external standard method. The linear regression equations, linear ranges, correlation coefficients, limits of detection (LOD), and limits of quantification (LOQ) for the three compounds are detailed in Table 3. All the calibration curves showed strong linearity over the tested range. The precision RSD values of the three control standards were lower than 2.28%, indicating that the precision of the method is acceptable. The RSD values for repeatability and stability were below 2.41%, indicating that the compounds remained essentially stable over a 24 h period. These results confirm the suitability of the method for the quantification of these compounds. The overall recoveries of the spiked samples ranged from 95.26% to 120%.

#### 2.7.2. Selection of Eluting Agents and Dosage

**Figure 8:**
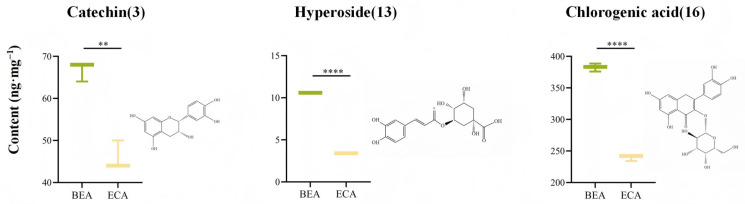
UHPLC-C-QTRAP-MS/MS was used to analyze the content of three representative components in BEA and ECA (values represented mean ± SEM ** *p* < 0.005, **** *p* < 0.0001).

UHPLC-C-QTRAP-MS/MS was used to determine the macroporous resin-extracted products of APL. The content of the components was calculated using the standard curve, and the mass spectrometry parameters are included in Table S5 of the Supplementary Material. The results shown in Table 3 indicate that the following components were mainly determined in the macroporous resin-extracted product of APL: catechin, hyperoside, and chlorogenic acid. As depicted in Figure 8, hyperoside had the highest average content (382.56 ± 3.65 ng·mg^−1^), followed by catechin (66.67 ± 1.33 ng·mg^−1^) and chlorogenic acid (10.60 ± 0.05 ng·mg^−1^). These three polyphenolic components were found to be significantly higher in BEA compared to ECA. In addition, the purity of catechin hydrate, hyperoside, and chlorogenic acid in BEA and ECA extraction products increased by 2.35%, 13.36%, and 0.29%, and 1.43%, 8.64%, and 0.11%, respectively.

### 2.8. Antiproliferative Activity of CT26 Tumor Cells and Wound-Healing Assay

**Figure 9:**
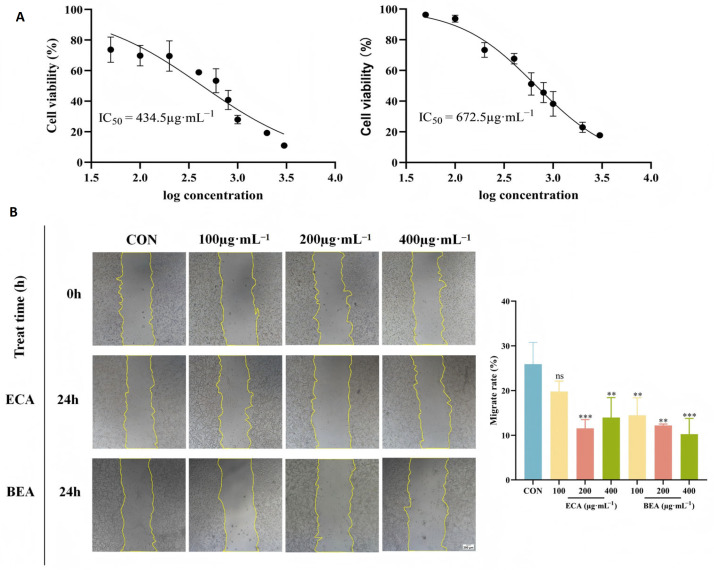
Cell viability and migration were inhibited by BEA and ECA extracts. (**A**) The cell viability of CT26 treated with BEA and ECA extracts was measured by using CCK8 assay. (**B**) CT26 tumor cells were exposed to different concentrations of BEA and ECA extracts for 24 h, and the migration ability was evaluated using a wound-healing test (*n* ≥ 3, values represented mean ± SD, ns for no significance, ** *p* < 0.01 vs. control, and *** *p* < 0.001).

The antiproliferative activity of CT26 tumor cells treated with BEA and ECA extracts at concentrations ranging from 50 to 3000 μg·mL^−1^ was evaluated using the CCK 8 assay. Figure 9A shows that the samples inhibited the proliferation of CT26 in a concentration-dependent manner. The IC_50_ values of BEA and ECA extracts were 434.50 and 672.5 μg·mL^−1^, respectively, which indicated that the antiproliferative activity of the BEA extract was stronger than that of ECA. It was hypothesized that the proliferative activity was related to a higher content of APLs that had been measured. Therefore, the two-step purification process of D101 resin combined with water-saturated extraction developed in this study can effectively improve the antiproliferative activity of APLs (data comparison before and after purification is shown in Table S6 of the Supplementary Material).

A wound-healing assay was performed to evaluate the lateral migration ability of the cells. Compared with the control group, the migration of BEA and ECA extracts was reduced; in comparison, between the two sites, the inhibition of lateral migration of CT26 tumor cells by BEA extracts was stronger at increasing concentrations, as shown in Figure 9B.

## 3. Discussion

D101 resin was the dominant resin for the enrichment of total polyphenols from APL. The results of the adsorption isotherm and kinetic fitting revealed that the pseudo-second-order kinetic model and the Langmuir isotherm model could better describe the adsorption process, and the pseudo-second-order kinetic model effectively described the adsorption behavior on D101 resin. In this study of adsorption thermodynamics, ΔG < 0 indicated that the adsorption process was spontaneous. The ΔH of the adsorption process was exothermic, and this result was consistent with that of adsorption kinetics. In addition, the absolute value of ΔH < 40 kJ·mol^−1^ indicates that the adsorption process is controlled by a physical mechanism rather than a chemical one. These findings suggest that the adsorption process is spontaneous and exothermic. In this study, the mechanism of adsorption of APLs on D101 macroporous resin was revealed, and the adsorption parameters and on-column elution process were optimized.

The total polyphenol content of the BEA extract (36.83%) was higher than that of the ECA extract (22.31%), and it was 2.63 times higher than that of the crude extract (see Table S5), which demonstrated the effectiveness of the purification process. The final optimal process was determined as follows: a 20 mg·mL^−1^, 4 BV solution of initial APLs was added to a D101 resin column at a specific rate (1 BV·h^−1^), and the D101 column was eluted with 5 BV of 70% ethanol, combined with the BEA extraction to obtain the APL macroporous resin-extracted product.

The liquid–mass spectrometry technique was used for the qualitative and quantitative analysis of the components in the macroporous resin-enriched products of APL. With reference to published mass spectrometry data, including retention times and fragmentation ion peaks, a total of 29 components, including catechin, hyperoside, and chlorogenic acid, were identified in BEA and ECA extracts. The results of the quantitative analysis showed that the contents of the components obtained from the two extraction sites were significantly different, with the levels of three polyphenolics, namely catechin, hyperoside, and chlorogenic acid, being significantly higher in the BEA extract than in the ECA.

In this experiment, pharmacodynamic validation was conducted for BEA and ECA extracts in order to identify the material basis for a more definite and effective treatment against colorectal cancer. Colorectal cancer, one of the most prevalent malignant tumors, is considered a dynamic process involving multi-level and multi-gene mutation accumulation and the interaction of many factors [42]. Antiproliferative activity and wound-healing experiments were carried out on CT26 tumor cells. The antiproliferative activity of the BEA extract was stronger than that of ECA, as demonstrated by the constructed corresponding tumor cell model. This conclusion was consistent with the results of the preliminary UHPLC-C-QTRAP-MS/MS results, and it was hypothesized that the difference in proliferative activity was related to variations in the content of APLs.

Ali et al. found that chlorogenic acid-induced apoptosis and cell cycle arrest in colorectal cancer cells by increasing the expression of P21 and P53 [43], while evidence from Huo showed that chrysin reduced the expression of Sirt1, thereby inhibiting tumor cell proliferation, invasion, and metastasis [44]. The results of Jung et al. showed that the n-butanol extract of APL could reduce the production of nitric oxide by inhibiting the expression of iNOS [45].

The anti-colorectal cancer activity of BEA and ECA extracts was concentration-dependent on polyphenols. It may also be that there is a synergistic effect between polyphenol monomers, which is based on complex molecular interactions that act on the same or different signaling pathways. These interactions may promote cancer cell apoptosis, inhibit angiogenesis, regulate the cell cycle, inhibit the release of inflammatory mediators and the production of ROS, and reduce oxidative stress, jointly inhibiting tumor growth [46,47].

## 4. Materials and Methods

### 4.1. Material and Reagents

APL purchased from Jiangsu Huahong Pharmaceutical Science and Technology Co., Ltd. (Zhengjiang, Jiangsu, China) (batch No. 240207), identified by Associate Professor Lisi Zou of Nanjing University of Traditional Chinese Medicine as the dried above-ground parts of *Agrimonia pilosa* Ledeb. D101, NKA-9, AB-8, and X-5 were purchased from Shanghai Yuanye Biotechnology Co., Ltd.(Shanghai, China) and SP-207 and 450 macroporous resins were purchased from Beijing Solebaum Technology Co., Ltd.(Beijing, China) (Table S1 lists the characteristics of the six macroporous resins). Gallic acid (J01IB218870), (+)-catechin hydrate (S01HB191501), hyperoside (A21IB223449), and chlorogenic acid reference standard (A22GB158496), with purity ≥98%, were purchased from Shanghai Yuanye Biotechnology Co., Ltd. (Shanghai, China) Folin-Phenol was obtained from Shanghai McLean Co., Ltd.(Shanghai, China). LC-MS-grade acetonitrile, methanol, and formic acid were purchased from Merck (Darmstadt, Germany). Ultrapure water was procured using the Milli-Q purification system (Millipore, Milford, MA, USA).

CT26 tumor cells were provided by the Jiangsu Collaborative Innovation Center for Tumor Prevention and Treatment with Traditional Chinese Medicine. RPMI-1640 medium, penicillin-streptomycin double antibiotic solution, and phosphate buffer salt were obtained from Shanghai Yuanpei Co., Ltd. in Shanghai, China. ABW Extra Fetal Bovine Serum was sourced from Shanghai Novartis Science and Technology Co., Ltd.(Shanghai, China), and the ultrasensitive cell proliferation assay reagent (CCK-8) was supplied by Wuhan Yacoyin Biotechnology Co., Ltd.(Wuhan, China)

### 4.2. Pretreatment of Macroporous Resins

The macroporous resin was soaked in anhydrous ethanol overnight, eluted with 95% ethanol until the effluent showed no white turbidity upon the addition of water, washed with ultrapure water until the effluent was free of alcoholic odor, washed and soaked for 2 h using 2 BV of 5% HCL solution and 2 BV of 2% NaOH solution in turn, and then washed with ultrapure water until the effluent was pH-neutral.

### 4.3. Extraction of Crude APL

APL powder (screened through the No. 4 sieve) was accurately weighed and subjected to triple reflux extraction with 70% ethanol at a solid–liquid ratio of 1:12. The resultant extract was concentrated under reduced pressure with simultaneous ethanol recovery. The concentrated solution was subsequently diluted with ultrapure water to achieve a final crude drug concentration of 0.1 g·mL^−1^.

### 4.4. Quantification of Total Polyphenols Content

Total polyphenol content was determined using the Folin–Ciocalteu assay. An appropriate volume of the test solution was transferred to a 10 mL volumetric flask, followed by the sequential addition of 0.5 mL of Folin–Ciocalteu reagent with 1 min of vortex mixing, and 2 mL of 20% sodium carbonate solution. The mixture was diluted to volume with ultrapure water and incubated for 2 h under light-protected conditions. Absorbance measurements at 750 nm were performed in triplicate, with gallic acid as the reference standard. The calibration curve exhibited linear regression (y = 10.621x + 0.0195, r^2^ = 0.9991), where x represents gallic acid concentration (mg·mL^−1^) and y denotes absorbance values.

### 4.5. Resin Screening

Approximately 1 g of each macroporous resin (AB-8, D101, X-5, SP-207, HPD-450, NKA-9) was transferred into a 50 mL stoppered conical flask. Each sample was combined with 20 mL of the APL solution and subjected to 24 h of orbital shaking (120 rpm) at room temperature. After filtration to remove the resin, the filtrate was analyzed for UV-Vis absorbance at 750 nm. Adsorption capacity, desorption quantity, and desorption ratio of APLs for each resin were calculated using the following equations:

(1) $$\begin{array}{c} q_{e}=\frac{V_{1}\left(C_{1}-C_{2}\right)}{m}, \end{array}$$

(2) $$\begin{array}{c} D_{e}\left(\%\right)=\frac{\left(C_{1}-C_{2}\right)}{C_{1}}, \end{array}$$

(3) $$\begin{array}{c} q_{d}=\frac{V_{2}C_{3}}{m}, \end{array}$$

(4) $$\begin{array}{c} D_{d}\left(\%\right)=\frac{V_{2}C_{3}}{V_{1}\left(C_{1}-C_{2}\right)}\times 100\%, \end{array}$$

where *q_e_* and *q_d_* are the adsorption equilibrium capacity (mg) and desorption capacity (mg), respectively. *C*_1_, *C*_2_, and *C*_3_ were the initial mass concentration (mg·mL^−1^), the mass concentration after macroporous resin adsorption (mg·mL^−1^), and the mass concentration after desorption (mg·mL^−1^) of total polyphenols from APL, respectively. *V*_1_ and *V*_2_ were the volumes of adsorption liquid and desorption liquid (mL), respectively, and *m* was the mass of the macroporous resin (g).

### 4.6. Adsorption Kinetics

APL extract (60 mL) with a crude drug content of 20 mg·mL^−1^ was placed in a 100 mL capped conical flask, and 3 g of pretreatment resin was added. Fully stirred at 25 °C and 120 rpm. Adsorption experiments were carried out at 0, 5, 10, 15, 30, 60, 90, 120, 180 and 240 min, respectively. Samples were taken at the corresponding time points, and the total polyphenol concentration was determined accordingly. The kinetic equations are as follows:

pseudo-first-order kinetic model equation:

(5) $$\begin{array}{c} ln\left(q_{e}-q_{t}\right)=\ln q_{e}-k_{1}t, \end{array}$$

pseudo-second-order kinetic model equation:

(6) $$\begin{array}{c} \frac{t}{q_{t}}=\frac{1}{k_{2}{q_{e}}^{2}}+\frac{t}{q_{e}}, \end{array}$$

particle diffusion model equation:
  (7) $$\begin{array}{c} q_{t}=k_{i}t^{\frac{1}{2}}+C_{i}, \end{array}$$
  where *q_t_* represents the adsorption capacity of the interval time *t* (mg·g^−1^ resin) and *q_e_* is the equilibrium adsorption per unit of resin (mg·g^−1^ resin); *k*_1_, *k*_2_, and *k*_i_ correspond to the first-order kinetic constant, the second-order kinetic constant and the particle diffusion kinetic constant, respectively. *C* is a constant in the particle diffusion model.

### 4.7. Adsorption Isotherm and Adsorption Thermodynamics

The extract of APL was diluted to 4, 8, 12, 16, and 20 mg·mL^−1^. Then 3 g of pretreated macroporous resin was weighed and placed in a 100 mL conical flask, and 60 mL of APL extract with different concentrations was added. Adsorption was performed at 25 °C, 35 °C, and 45 °C with 100 rpm oscillation for 24 h. The total polyphenol concentration in the supernatant was determined to calculate the adsorption amount.

In order to explain the adsorption mechanism, the adsorption isotherms of APLs on D101 macroporous resin were plotted by combining Langmuir, Freundlich, and Temkin [35,36,48] models. The thermodynamic equations are as follows:

Langmuir equation:

(8) $$\begin{array}{c} q_{e}=\frac{Q_{m}K_{L}C_{e}}{1+K_{L}C_{e}}, \end{array}$$

Freundlich equation:

(9) $$\begin{array}{c} lnq_{e}=\frac{1}{n}\ln C_{e}+\ln K_{F}, \end{array}$$

Temkin equation:

(10) $$\begin{array}{c} q_{e}=B_{T}lnK_{T}+B_{T}\ln C_{e}, \end{array}$$

where *q_e_* is adsorption capacity at equilibrium (mg·g^−1^), *K_L_* is a Langmuir association—a larger *K_L_* value indicates stronger adsorption performance of the adsorbent; *q_m_* is the maximum theoretical adsorption capacity (mg·g^−1^ resin); *n* and *K_F_* (mg·g^−1^) are Freundlich constants; *K_F_* is the theoretical saturated adsorption capacity; and *K_L_* and *B_T_* represent the constants of the Temkin isotherm model;

Gibbs free energy:

(11) $$\begin{array}{c} ∆G=-RTlnK_{c}, \end{array}$$

Enthalpy change:

(12) $$\begin{array}{c} lnK_{C}=\frac{∆S}{R}-\frac{∆H}{R_{T}}, \end{array}$$

(13) $$\begin{array}{c} ∆S=\frac{∆H-∆G}{T}, \end{array}$$

where *R* is the universal gas constant, and *T* is the absolute temperature. The values of Δ*H* are calculated from the slope and intercept of *lnC_e_* versus the 1/*T* curve.

### 4.8. Dynamic Adsorption and Desorption

#### 4.8.1. Dynamic Leakage Curve

The APL extract was diluted to a crude drug concentration of 20 mg·mL^−1^ and centrifuged at 10,000 rpm for 10 min at room temperature to obtain the column liquid. The pretreated D101 macroporous resin 3 g (wet mass, column volume 3 mL) was loaded into a chromatographic column (13 mm × 400 mm) using wet packing. The resin was washed with ultrapure water until the effluent had no alcohol odor. The upper column liquid was added slowly, and the effluent was collected at a flow rate of 1 BV·h^−1^.

#### 4.8.2. Selection of Eluting Agents and Dosage

Approximately 3 g of the pretreated D101 macroporous adsorption resin was slowly loaded into the adsorption column using wet packing. The crude drug concentration of 20 mg·mL^−1^ APLs extract was centrifuged at 25 °C (10,000 rpm, 10 min), and the supernatant was taken. The sample was loaded at a flow rate of 1 BV·h^−1^, the filtrate was collected, and the resin was allowed to adsorb for 2 h. After the adsorption was saturated, it was washed with ultrapure water until the effluent became colorless. Elution was performed using different ethanol at different concentrations to elute the target compound. The optimal elution solvent was determined through the above steps, and the elution volume was checked.

### 4.9. Liquid–Liquid Extraction Test

In order to better enrich and purify APLs, the macroporous resin-enriched products of APL, after purification with macroporous resin, were successively extracted with ethyl acetate and water-saturated n-butanol (at a material-to-liquid of 1:1) to obtain the macroporous resin-extracted products of BEA and ECA. The organic reagents were removed by vacuum concentration and redissolved with ultrapure water.

### 4.10. Macroporous Resin-Based Qualitative and Quantitative Analysis of APL Extraction Products

#### 4.10.1. Preparation of Standard Solutions

The appropriate amount of the three standard substances was accurately weighed, dissolved in methanol, and diluted in a 10 mL volumetric flask. The concentration of each standard substance was 0.10 mg·mL^−1^, and the mixed concentration was 200 ng·mL^−1^. The standard curve was prepared using the gradient dilution method.

#### 4.10.2. Sample Preparation

The appropriate amounts of BEA and ECA extracts were centrifuged (10,000 rpm, 10 min). The supernatant was diluted with methanol to a crude drug concentration of 0.1 mg·mL^−1^, filtered, and the filtrate was collected.

In order to evaluate the anti-colorectal cancer activity, the BEA and ECA extracts were freeze-dried using the above preparation method, weighing 3.57 g and 1.73 g, respectively.

#### 4.10.3. Characterization of the Extraction Products from the Macroporous Resin of APL

Peak View 1.2 (AB Sciex, Foster City, CA, USA) was used to analyze the data obtained by UPLC-Q-Zeno-TOF-MS/MS, and the ion current diagram (BPC) of APL macroporous resin-extracted product in negative ion mode was obtained. The chromatographic column used was a Waters ACQUITY UHPLC BEH C18 column (2.1 × 100 mm, 1.7 μm) (Waters Corporation, Drinagh, Ireland) Wexford, with the temperature maintained at 30 °C. The mobile phase was composed of solvent A (acetonitrile) and solvent B (water containing 0.1% formic acid). The flow rate was set at 0.3 mL·min^−1^, the injection volume was 1 μL, and the gradient elution was set to 0–7 min, 5–20% A; 7–13 min, 20–25% A; 13–17 min, 25–40% A; 17–26 min, 40–60% A; and 26–35 min, 60–95% A.

The electrospray ion source (ESI) was used to scan in positive and negative ion modes. The spray voltage was 5500 eV, and the ion source temperature was 550 °C. The atomizing gas, auxiliary gas, and curtain gas were all high-purity nitrogen. The pressure of the atomizing gas (GS1) and the auxiliary gas (GS2) was 55 pa. The curtain gas (CUR) was 35 mL·min^−1^, the declustering voltage (DP) was 70 eV, the collision voltage (CE) was 45 eV, and the collision voltage difference (CES) was 15 eV.

#### 4.10.4. UHPLC-C-QTRAP-MS/MS-Based Quantitative Analysis of the Macroporous Resin-Extracted Products of APL

The chromatographic conditions adhered to those outlined in Section 4.10.3, with the gradient elution set as follows: 0–0.5 min, 5–5% A; 0.5–2 min, 5–25% A; 2–6 min, 25–40% A; 6–8 min, 40–60% A; 8–10 min, 60–95% A; 10–12 min, 95–95% A; 12–12.5 min, 95–5% A; 12–15 min, 5% A. Quantitative analysis of representative compounds was performed using a triple quadrupole liquid chromatography–mass spectrometer (Triple Quad6500^+^ system), and the data were processed using SCIEX OS software 2.1.

### 4.11. Antiproliferative Activity of CT26 Tumor Cells

CT26 tumor cells in a logarithmic growth phase were selected and diluted to contain 3 × 10^4^ cells per milliliter. The diluted cell suspension was evenly added to a 96-well plate, and 100 μL of medium was added to each well. 

The blank group, control group, ECA group, and BEA group were set up. After the cells adhered to the wall, the corresponding drugs (crude drug concentrations of 0, 0.05, 0.1, 0.2, 0.4, 0.6, 0.8, 1, 2, and 3 mg·mL^−1^) were added to each group and cultured in a 5% CO_2_ incubator at 37 °C for 24 h. Three repeated groups were established in each category. After the drug intervention, 10 μL of CCK-8 solution was added to each well. After 2 h of culture, the absorbance at 450 nm was measured using a microplate reader to assess cell viability.

### 4.12. Wound-Healing Assay

CT26 tumor cells in a logarithmic growth state were inoculated in a six-well plate at 5 × 10^5^ cells/well and cultured in a 5% CO_2_ incubator at 37 °C for 24 h. The blank group, BEA group, and ECA group were set up. After the cells were full (when the degree of fusion reached 95%), a 10 μL sterile pipette tip was used to draw a straight line perpendicular to the back of the plate. After washing with PBS to remove the cell debris, the cells were administered at low, medium, and high concentrations (100, 200, and 400 μg·mL^−1^). Scratch wound images were taken and recorded at 0 and 24 h. Three visual fields were recorded per well at each time point, and the cell migration area was measured. ImageJ software (https://imagej.net/ij/) was used to determine the wound area by drawing the boundary around the wound area to obtain a pixel count. The scratch migration rate = (A_n_ − A_0_)/A_0_ × 100%, where A_0_ represents the scratch area immediately after scratching at 0 h, and A_n_ is the scratch area after scratching for 24 h. The data were expressed as the average of three copies, and the error was expressed as the standard deviation.

## 5. Conclusions

A tumor is a multi-factor interactive disease, and treatment requires multi-pathway and multi-target coordination. APL is rich in polyphenols, which can be divided into four categories: phenolic acids, flavonoids, lignans and stilbenes [49]. Modern studies have confirmed that these polyphenols have various activities, such as inducing tumor cell apoptosis, inhibiting angiogenesis, and regulating immune function, which are of positive significance for the multi-target treatment of tumors [50,51]. Based on the properties of these components, this study determined and optimized a macroporous resin-coupled extraction method, analyzed the composition of the extracts and the intensity of their anti-tumor effect in vitro, and provided a scientific basis for the further development of new drugs derived from these components.

## Figures and Tables

**Table 1:** Adsorption thermodynamic parameter results of total polyphenols.

T (K)			ΔS (J·mol^−1^)	ΔH (KJ·mol^−1^K)
ΔG (kJ·mol^−1^)				
298	308	318		
−0.90	−0.77	−0.54	−10.84	−4.09

**Table 2:** Compounds data identified from APL according to UPLC-Q-Zeno-TOF-MS/MS.

NO.	R (min)	Identification	Molecular Formula	MW	Measured (*m*/*z*)	Characteristic Fragment Ions	Error (ppm)	
1	2.47	3-Hydroxy-4-methoxybenzoic acid	C_8_H_8_O_4_	167.0350	167.0362	163.0303, 152.0115, 123.0437, 108.0217	1.7	[M-H]^−^
2	2.85	Protocatechuic acid	C_7_H_6_O_4_	153.0193	153.0203	109.0301, 108.6876, 108.0213, 91.0205, 81.0356	6.4	[M-H]^−^
3	4.4	Catechin	C_15_H_14_O_6_	289.0718	289.0724	135.0453, 151.0397, 179.0341, 187.0394, 203.0721, 245.0821, 271.0603	2.4	[M-H]^−^
4	5.02	Caffeic acid	C_9_H_8_O_4_	179.0350	179.0357	135.0439, 134.0360, 117.0358, 89.0417	4.1	[M-H]^−^
5	5.04	Procyanidin B3	C_30_H_26_O_12_	577.1351	577.1346	451.1023, 425.0867, 407.0767, 287.0543, 289.0710, 161.0235, 125.0242	−1	[M-H]^−^
6	5.72	(1R,3S,4R,5S)-1,3,4-trihydroxy-5-[(E)-3-(4-hydroxyphenyl)prop-2-enoyl]oxy-cyclohexane-1-carboxylic acid	C_16_H_18_O_8_	338.1002	337.0929	173.0463, 163.0410, 155.0355	0.8	[M-H]^−^
7	6.29	4-O-Feruloylquinic acid	C_17_H_20_O_9_	368.1107	367.1035	193.0495, 191.0569, 149.0603, 134.0371, 117.0340	0	[M-H]^−^
8	6.69	p-Coumaric acid	C_9_H_8_O_3_	164.0473	163.0401	119.0661, 119.0482, 92.9962	7.1	[M-H]^−^
9	6.85	Gallic acid	C_7_H_6_O_5_	169.0143	169.0152	125.0235, 123.0071, 107.0192, 95.0112, 79.0184	5.3	[M-H]^−^
10	6.97	Dehydrodicatechin A	C_30_H_24_O_12_	575.1195	575.1193	575.1215, 449.0933, 394.0695, 309.0075, 271.0235, 229.0492, 161.0222, 137.0230	−0.2	[M-H]^−^
11	7.3	Ellagic acid	C_14_H_6_O_8_	300.9990	300.9991	283.9954, 257.0068, 229.0128, 185.0248	0.4	[M-H]^−^
12	7.45	Rutin	C_27_H_30_O_16_	610.1539	610.1497	447.0893, 413.0954, 300.0516, 300.0263, 271.0226, 151.0040	0	[M-H]^−^
13	7.64	Hyperoside	C_21_H_20_O_12_	463.0882	463.0878	301.0357, 300.0275, 271.0248, 255.0302, 243.0303, 178.9988, 151.0036	−0.9	[M-H]^−^
14	7.85	Kaempferol	C_15_H_10_O_6_	285.0405	285.0408	217.0491, 198.0295, 175.0426, 151.0041, 133.0297, 132.0214, 121.0280, 107.0153	1.1	[M-H]^−^
15	7.87	Taxifolin	C_15_H_12_O_7_	303.0510	303.0516	285.04808, 275.0511, 217.0514, 199.0361, 175.0379, 153.0182, 125.022	0.9	[M-H]^−^
16	8.9	Chlorogenic acid	C_16_H_18_O_9_	353.0878	353.0882	135.0450, 161.0246, 173.0244, 179.0349, 191.0560	0.8	[M-H]^−^
17	8.97	Quercitrin	C_21_H_20_O_11_	447.0933	447.0934	301.0355, 300.0277, 271.0252, 255.0304, 151.0038	0.2	[M-H]^−^
18	9.67	Dihydrokaempferol	C_15_H_12_O_6_	287.0561	287.0567	287.0783, 259.0572, 243.0669, 177.0544, 155.0511, 152.0092, 125.0247, 106.0428	0.5	[M-H]^−^
19	9.82	Isochlorogenic acid B	C_25_H_24_O_12_	516.1268	515.1195	447.1014, 353.0903, 255.0770, 191.0561, 179.0344, 173.0462, 135.0423	−0.6	[M-H]^−^
20	9.97	Dihydrocaffeic acid	C_9_H_10_O_4_	182.0579	181.0506	153.0179, 136.9219, 111.0065, 109.0294, 108.0211, 107.0155	6.5	[M-H]^−^
21	10.62	Cynaroside	C_21_H_20_O_10_	431.0984	431.0983	229.0503, 227.0346, 255.0299, 285.0403, 284.0323, 431.0974	0.2	[M-H]^−^
22	12.02	Isochlorogenic acid A	C_25_H_24_O_12_	516.1268	515.1195	173.0495, 353.0903, 173.0457, 179.0344, 191.0561, 135.0423, 179.00540	−0.6	[M-H]^−^
23	13.49	Quercetin	C_15_H_10_O_7_	301.0358	301.0356	283.9909, 257.0407, 219.0657, 178.9984, 151.0032, 121.0292	−0.6	[M-H]^−^
24	14.32	Tiliroside	C_30_H_26_O_13_	593.1301	593.1306	447.0919, 307.0814, 285.0392, 284.0314, 255.0299, 227.0339	0.9	[M-H]^−^
25	15.87	Apigenin	C_15_H_10_O_5_	269.0455	269.0463	269.0451, 225.0536, 151.0045, 121.0260, 117.0348, 107.0128	−0.5	[M-H]^−^
26	16.09	Kaempferide	C_16_H_12_O_6_	299.0561	299.0557	284.0319, 299.0558	−1.9	[M-H]^−^
27	16.29	Luteolin	C_15_H_10_O_6_	285.0405	285.0408	198.0295, 175.0426, 151.0041, 133.0297	−1.6	[M-H]^−^
28	17.35	Agrimonolide-6-O-glucopyranoside	C_24_H_28_O_10_	475.1610	475.1603	313.1080, 312.8826, 298.0841, 148.0532	−2	[M-H]^−^
29	33.31	Agrimol B	C_37_H_46_O_12_	681.2917	681.2952	653.3028, 447.1237, 285.1277, 116.9352	5.2	[M-H]^−^

**Table 3:** Linear relationships, limits of detection (LOD), and limits of quantitation (LOQ) for three analytes obtained using UHPLC-C-QTRAP-MS/MS, including the quantification of ECA and EBA.

Analyte	Linearity			LOD (ng·mL^−1^)	LOQ (ng·mL^−1^)	Content (ng·mg^−1^)	
	Calibration Curve	Range (ng·mL^−1^)	r^2^			BEA	ECA
Catechin (3)	Y = 1849.5x − 3145.2	3.37–200.73	0.9977	1.78	3.37	66.67 ± 1.33	46.00 ± 2.00
Hyperoside (13)	Y = 22,989x − 12,386	1.15–199.54	0.9980	0.80	1.15	382.56 ± 3.65	239.40 ± 2.60
Chlorogenic acid (16)	Y = 6655.7x − 5239	1.94–198.5	0.9988	1.04	1.94	10.60 ± 0.05	3.42 ± 0.01

## Data Availability

The data used to support the findings of this study are available from the corresponding author upon request.

## Supplementary Materials

The following supporting information can be downloaded at https://www.mdpi.com/article/10.3390/molecules30071478/s1, Figure S1: The characteristic ion and its corresponding secondary mass spectral information of catechin. Table S1: Characteristics of six kinds of macroporous resins; Table S2: Correlation coefficients of pseudo-first-order, pseudo-second-order and intra-particle diffusion models; Table S3: The correlation coefficient of the three models; Table S4: The corresponding structures and CAS numbers of 29 compounds; Table S5: Mass spectrometry parameters of chlorogenic acid, hyperoside and catechin; Table S6: Relevant data before and after purification of total polyphenols.
