# Supplementary material for: Analysis of Macroporous Resin Combined Extraction and Purification of Polyphenols from Agrimonia pilosa Ledeb. and Anti-Tumor Effect In Vitro
Source: Molecules. 2025 Mar 26;30(7):1478. doi: 10.3390/molecules30071478 (PMC11990265; doi:10.3390/molecules30071478)
Supplement: Supplementary file 1 [file molecules-30-01478-s001.zip › Supplementary Materials Fig. S1, Table S1-S3 and S5-S6.pdf]

Supplementary Material

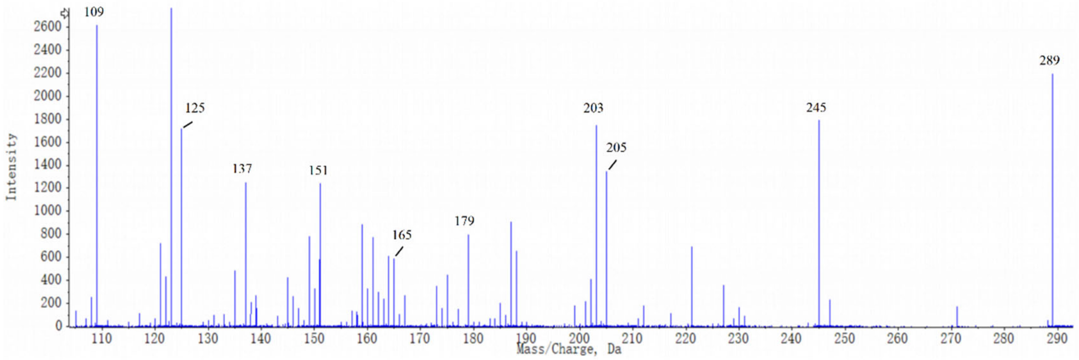

Figure S1. The characteristic ion and its corresponding secondary mass spectral information of catechin.

Table S1. Characteristics of 6 kinds of macroporous resins.

Resin Variety	Average Pore Size(nm)	Polarity	Specific Surface Area
NKA-9	150-170	Middle polar	250-290
HPD-450	90-100	Middle polar	≥500
AB-8	130-140	Weak-polar	450-530
D101	90-100	Weak-polar	550-600
SP-207	110	Non-polar	600
X-5	290-300	Non-polar	500-600

Table S2. Correlation coefficients of pseudo-first-order, pseudo-second-order and intraparticle diffusion models.

Kinetics model	Equations	Dynamic parameters
Intra-particle diffusion	$q_t = 0.1808\ t^{1/2} + 2.766$	k_i=0.1808<br>C=3.0675<br>R^2=0.8018
Pseudo-first-order	$\ln (q_e\text{-}q_t) = -0.0050\ t + 0.9373$	k_1=0.0050<br>q_1=6.8944<br>R^2=0.7235

Pseudo-second-order	$t/q_t = 0.9951\ t + 6.6243$	k_2=0.0047<br>q_2=6.6243<br>R^2=0.9951

Table S3. The correlation coefficient of the three models.

Temperature (℃)	Langmuir Equation			Freundlich Equation			Temkin model		
	q_m	K_L	R^2	K_F	1/n	R^2	B_T	K_T	R^2
25	8.7298	0.4616	0.9726	2.2565	0.6236	0.9883	1.6743	4.1020	0.9486
35	6.8162	0.4123	0.9851	2.0634	0.5953	0.9866	1.5457	3.9937	0.9736
45	5.6999	0.4027	0.9574	1.8261	0.5481	0.9679	1.4498	3.6409	0.9243

Table S5 Mass spectrometry parameters of chlorogenic acid, hyperoside and catechins.

ingredient	ion pair	DP	CE
Chlorogenic Acid	353→191.2	-20	-29
Hyperoside	463.1→300.1	-80	-39
Catechin	289→245	-80	-14

Table S6. Relevant data before and after purification of total polyphenols.

	Crude extract	The macroporous resin-extracted products
Total Polyphenol Content	22.51%	59.14%
Inhibiting the IC50 of CT-26 cell line （μg/mL）	1414	672.5* 434.5#

* represented ECA，# represented BEA
