# Supplementary material for: Analysis of Macroporous Resin Combined Extraction and Purification of Polyphenols from Agrimonia pilosa Ledeb. and Anti-Tumor Effect In Vitro
Source: Molecules. 2025 Mar 26;30(7):1478. doi: 10.3390/molecules30071478 (PMC11990265; doi:10.3390/molecules30071478)

Supplementary Material

S4 The corresponding structures and CAS numbers of 29 compounds.

1. 3-Hydroxy-4-methoxybenzoic acid CAS: 645-08-9

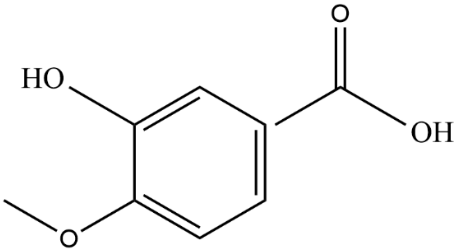

2. Protocatechuic acid CAS: 99-50-3

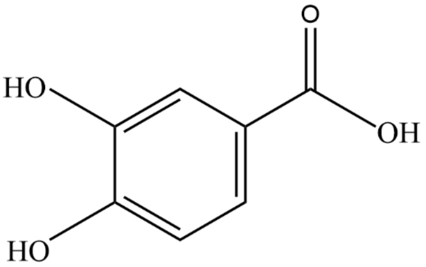

3. Catechin CAS: 18829-70-4

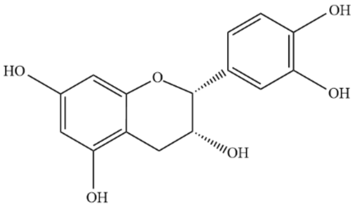

4. Caffeic acid CAS: 331-39-5

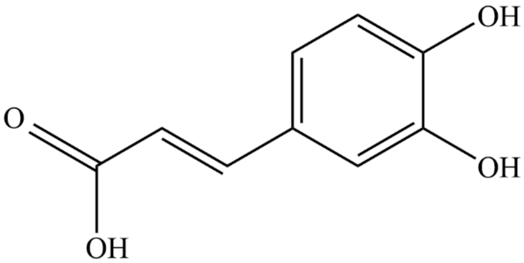

5. Procyanidin B3 CAS: 23567-23-9

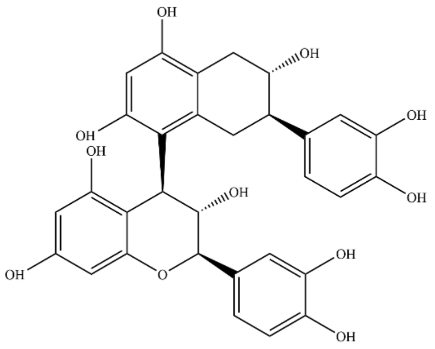

6. (1R,3S,4R,5S)-1,3,4-trihydroxy-5-[(E)-3-(4-hydroxyphenyl)prop-2-enoyl]oxy-cyclohexane-1-carboxylic acid CAS: 5746-55-4

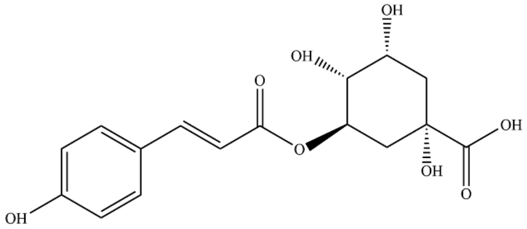

7. 4-O-Feruloylquinic acid CAS: 2613-86-7

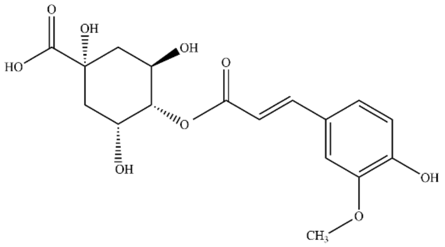

8. p-Coumaric acid CAS: 501-98-4

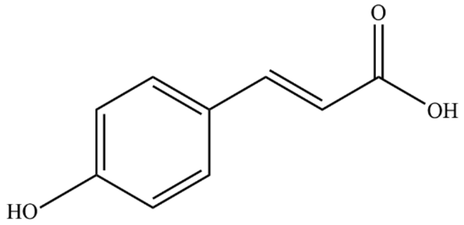

9. Gallic acid CAS: 149-91-7

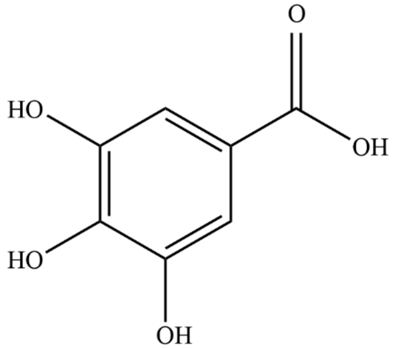

10. Dehydrodicatechin A CAS: 36048-23-4

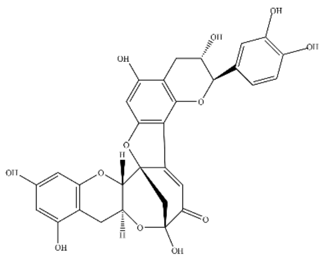

11. Ellagic acid CAS: 476-66-4

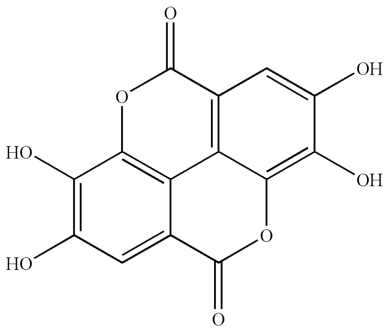

12. Rutin CAS: 153-18-4

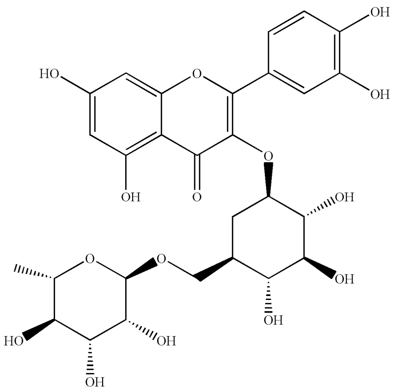

13. Hyperoside CAS: 482-36-0

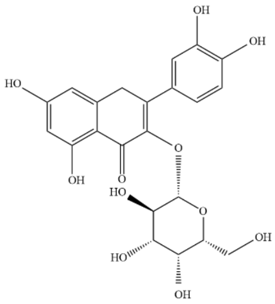

14. Kaempferol CAS: 520-18-3

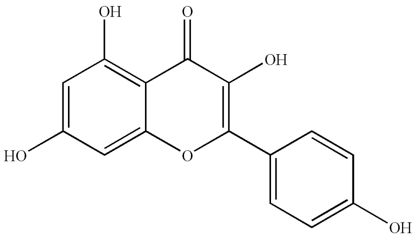

15. Taxifolin CAS: 480-18-2

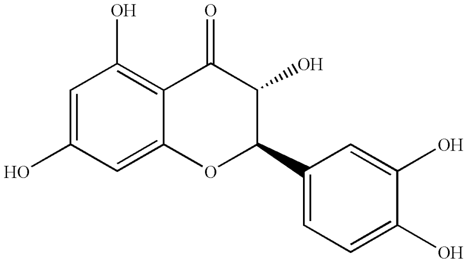

16. Chlorogenic acid CAS: 327-97-9

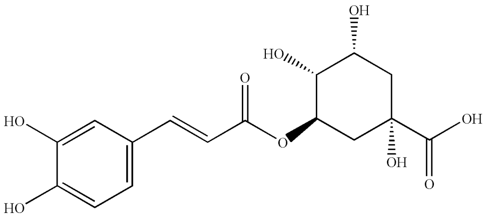

17. Quercitrin CAS: 522-12-3

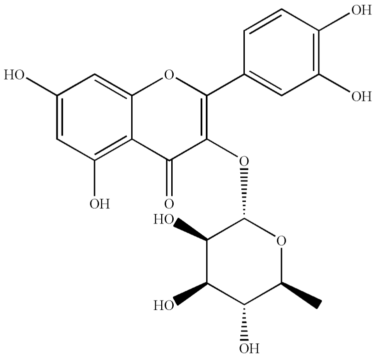

18. Dihydrokaempferol CAS: 480-20-6

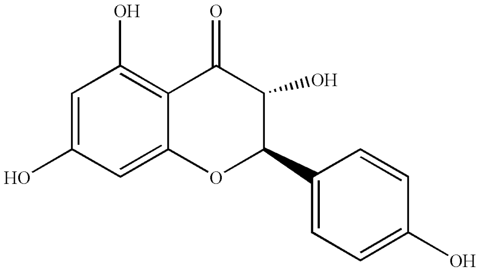

19. Isochlorogenic acid B CAS: 14534-61-3

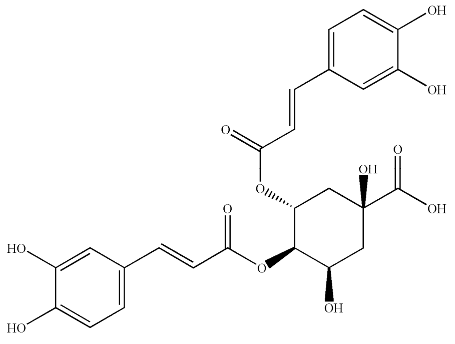

20. Dihydrocaffeic acid CAS: 1078-61-1

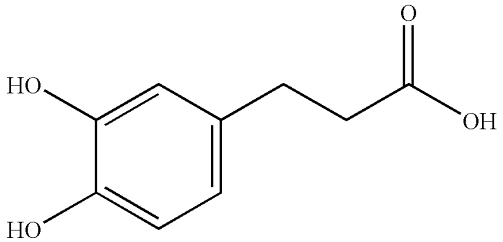

21. Cynaroside CAS:5373-11-5

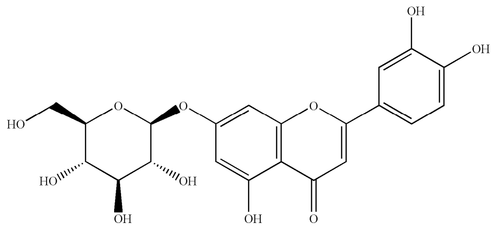

22. Isochlorogenic acid A CAS: 2450-53-5

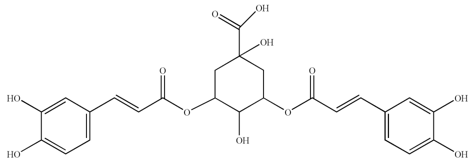

23. Quercetin CAS: 117-39-5

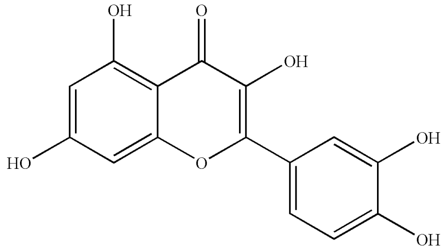

24. Tiliroside CAS: 20316-62-5

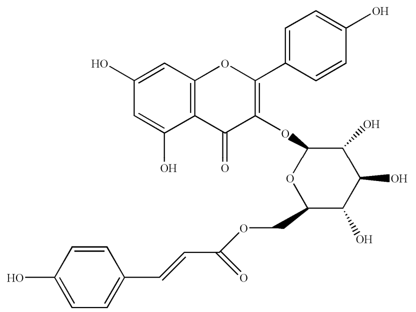

25. Apigenin CAS: 520-36-5

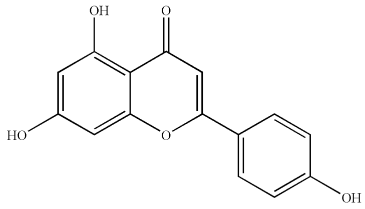

26. Kaempferide CAS: 491-54-3

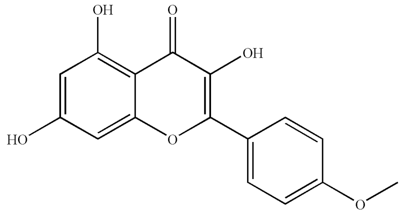

27. Luteolin CAS: 491-70-3

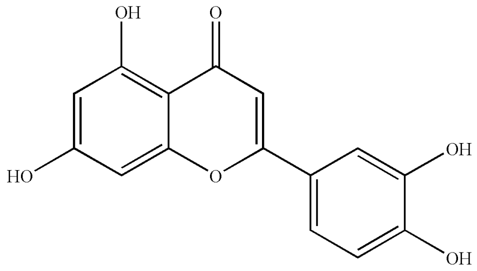

28. Agrimonolide-6-O-glucopyranoside CAS: 126223-29-8

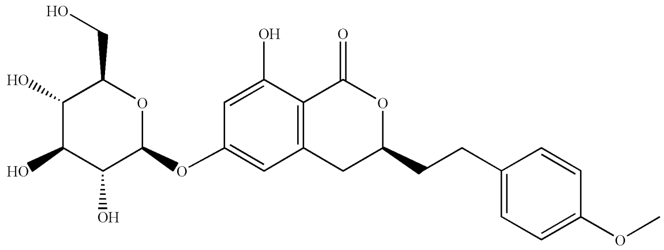

29. Agrimol B CAS: 55576-66-4

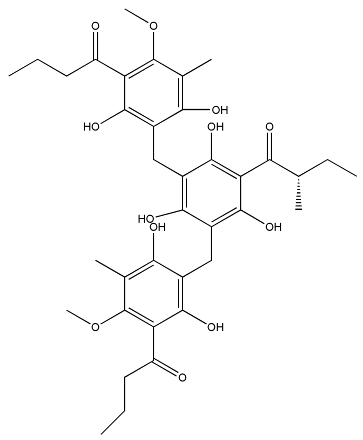

Supplement: Supplementary file 1 [file molecules-30-01478-s001.zip › Supplementary Materials Table S4.pdf]
